# Behavioral variant frontotemporal neurocognitive disorder or psychiatric disorder: a diagnostic challenge

**DOI:** 10.1192/j.eurpsy.2025.1871

**Published:** 2025-08-26

**Authors:** M. A. Sava, S. P. Orrego Molina, M. D. S. Manzano Palomo, P. Á. Nava García, A. M. Durán Moreno

**Affiliations:** 1 Psychiatry; 2 Neurology, Hospital Universitario Infanta Leonor; 3 Psychiatry, Hospital Universitario de Fuenlabrada, Madrid, Spain

## Abstract

**Introduction:**

The behavioral variant of frontotemporal neurocognitive disorder is the most common clinical syndrome within frontotemporal neurocognitive disorders and is primarily characterized by progressive changes in personality and behavior. Due to the heterogeneity of this condition and the lack of sensitive and specific complementary tests, patients are often misdiagnosed with a psychiatric disorder.

**Objectives:**

To describe the clinical case of a patient with a long-standing history of psychiatric pathology who was admitted to the short-stay psychiatric unit due to newly emerged behavioral disturbances, and to conduct a brief review of the literature.

**Methods:**

Case description and literature review.

**Results:**

A 57-year-old male under psychiatric follow-up for Obsessive-Compulsive Disorder, impulse control disorder (kleptomania), and anxiety-depressive symptoms was voluntarily admitted to the short-stay psychiatric unit due to behavioral disturbances. During admission, the patient reported emotional dysregulation, experiencing irritability and difficulties with emotional containment and inhibition, noting feelings of impulsive discontrol and behavioral disinhibition. The family observed changes in the patient’s personality over the past months, describing him as aggressive and inappropriate.

Basic complementary tests (blood analysis, urine analysis, and toxicology screening) showed no significant alterations. Brain MRI revealed subtle signs of cortico-subcortical atrophy, predominantly in the frontotemporal regions. Cognitive assessments, including the Mini Mental State Examination, Frontal Assessment Battery and Montreal Cognitive Assessment, indicated deficits suggestive of frontal impairment. The patient had been admitted one month prior due to possible hypomanic symptoms that resolved following pharmacological adjustment, leading to a proposed diagnosis of late-onset Bipolar Disorder Type II. However, after detailed history-taking with family members and additional testing, the diagnosis leaned more towards a frontal syndrome (possible behavioral variant of frontotemporal neurocognitive disorder).

**Conclusions:**

The diagnosis of frontotemporal neurocognitive disorder remains a significant challenge, partly due to the limitations of complementary tests in the early stages of the disease, with diagnosis primarily based on clinical evaluation. Consequently, many patients with frontotemporal neurocognitive disorder initially receive a diagnosis of a psychiatric condition (more frequently if the patient has a personal history of psychiatric issues). Therefore, it is crucial to understand these differences, especially in the early phases of the disease, and to continue investigating new diagnostic modalities.

**Disclosure of Interest:**

None Declared

